# Inferring RNA sequence preferences for poorly studied RNA-binding proteins based on co-evolution

**DOI:** 10.1186/s12859-018-2091-8

**Published:** 2018-03-12

**Authors:** Shu Yang, Junwen Wang, Raymond T. Ng

**Affiliations:** 1 Department of Computer Science, University of British Columbia, Vancouver, Canada; 2 Department of Health Sciences Research, Mayo Clinic Arizona, Scottsdale, USA

**Keywords:** RBP binding preference, K-nearest neighbors, Co-evolution, Machine learning

## Abstract

**Background:**

Characterizing the binding preference of RNA-binding proteins (RBP) is essential for us to understand the interaction between an RBP and its RNA targets, and to decipher the mechanism of post-transcriptional regulation. Experimental methods have been used to generate protein-RNA binding data for a number of RBPs in vivo and in vitro. Utilizing the binding data, a couple of computational methods have been developed to detect the RNA sequence or structure preferences of the RBPs. However, the majority of RBPs have not yet been experimentally characterized and lack RNA binding data. For these poorly studied RBPs, the identification of their binding preferences cannot be performed by most existing computational methods because the experimental binding data are prerequisite to these methods.

**Results:**

Here we propose a new method based on co-evolution to predict the sequence preferences for the poorly studied RBPs, waiving the requirement of their binding data. First, we demonstrate the co-evolutionary relationship between RBPs and their RNA partners. We then present a K-nearest neighbors (KNN) based algorithm to infer the sequence preference of an RBP using only the preference information from its homologous RBPs. By benchmarking against several in vitro and in vivo datasets, our proposed method outperforms the existing alternative which uses the closest neighbor’s preference on all the datasets. Moreover, it shows comparable performance with two state-of-the-art methods that require the presence of the experimental binding data. Finally, we demonstrate the usage of this method to infer sequence preferences for novel proteins which have no binding preference information available.

**Conclusion:**

For a poorly studied RBP, the current methods used to determine its binding preference need experimental data, which is expensive and time consuming. Therefore, determining RBP’s preference is not practical in many situations. This study provides an economic solution to infer the sequence preference of such protein based on the co-evolution. The source codes and related datasets are available at https://github.com/syang11/KNN.

**Electronic supplementary material:**

The online version of this article (10.1186/s12859-018-2091-8) contains supplementary material, which is available to authorized users.

## Background

Determining the binding preference of an RBP is central to investigating RNA-protein interactions. Such preference, also known as specificity, denotes the RBP’s preferential association with specific RNA sequence motifs (i.e. sequence preference) or structure motifs (i.e. structure preference) [1]. Typically, in order to characterize the preference of an RBP, experimental methods are designed to generate binding data consisted of enriched RNA sequences bound by a particular RBP, either in vivo like CLIP (Crosslinking immunoprecipitation) based method (HITSCLIP, PAR-CLIP and iCLIP) [2, 3] or in vitro like RNAcompete assays [4, 5]. Computational methods are then used to predict a binding model pertaining to that RBP based on the binding data.

However, due to the limited availability of experimental data, only a small fraction of the RBPs from a few representative species have been well studied regarding their preferences up to now. Identifying the RNA targets bound by novel or poorly studied RBPs remains a challenge. Currently, most experimental methods employ microarray [4] or next-generation sequencing [6] to assay the corresponding RNA sequence information of an RBP. Although there are methods such as icSHAPE [7] that can determine RNA structures, RNA structure data is not captured in most experimental methods, and it is usually predicted from sequence data using algorithms such as RNAshapes [8] and RNAplfold [9]. Given the experimental data as input, a number of computational methods have been developed to build binding preference models. Those methods can be roughly classified into two categories: (1) methods focusing on sequence models, i.e. considering RNA sequence information alone for binding preference [10–12]; (2) methods focusing on sequence and structure models, i.e. considering both RNA sequence and structure information for binding preference [13–17]. Some representative methods are summarized in Table 1.

**Table 1:** Representative computational methods for RBP binding preference prediction

Method	Input data	Ref	Highlight
DeepBind	RNAcompete	[10]	Learning sequence preference as
			the convolution function in a deep
			convolutional neural network
MEMERIS	SELEX	[13]	Estimating sequence preference
			(PWM) with single-stranded
			structure context by maximum
			likelihood estimation
Li et al.	RIP-chip	[14]	Predicting sequence preference
			(consensus) with single-stranded
			structure context by iterative
			refinement
RNAcontext	RNAcompete	[15]	Learning a joint model with
			PWM for sequence preference
			and probability vector for structure
			preference
GraphProt	CLIP-seq	[16]	Learning sequence and structure
			preference using graph encoding
			and graph-kernel SVM
RCK	RNAcompete	[17]	Extending RNAcontext using
			position-dependent k-mer model
			for sequence and structure
			preference

For an RBP of interest, all the methods in Table 1 require the RBP’s experimental binding data as input to directly determine the preference. We call these methods “direct” methods to distinguish them from “inferred” methods that predict the preference indirectly from other RBPs with known preferences. The latter category is the focus of this paper. The binding preference of a novel or poorly studied RBP that only has amino acid sequence available could not be predicted by any of the “direct” methods. To the best of our knowledge, only one study has suggested an “inferred” workaround for such case [5]. As observed by Ray et al. in this study, RBPs that have identity > 70% in their RNA-binding domain sequences have similar target RNA sequence motifs. Hence, the authors assumed that the sequence preference (represented by position weight matrix (PWM) [18]) of a poorly studied RBP would be the same as a well-studied RBP if more than 70% of the sequences within their RNA-binding domains are identical. Based on this assumption, Ray et al. inferred sequence preferences for poorly studied RBPs across 288 sequenced eukaryotes. These binding preferences were deposited into the cisBP-RNA database [19]. Nevertheless, this inference only provides a crude estimation, and could not work for RBPs that do not have highly homologous RBPs. In spite of the obvious limitations of this method, it implies the conserved correlation between RBP sequences and their RNA binding targets along evolution.

In this paper we introduce a machine learning approach to predict the sequence preference for poorly studied RBPs. The proposed approach is an “inferred” method that utilizes co-evolution between the RBPs and their binding RNAs. The use of co-evolution has not yet been explored between the RBPs and their binding RNAs, although it has been widely studied in protein-protein interactions [20, 21] and DNA-protein interactions [22, 23]. In general, mutations in either the RBP or the RNA target may weaken their interactions, potentially leading to abnormality in organisms. In fact, a number of diseases have been previously reported to be linked to the mis-regulation or malfunction of specific RNA-protein interactions [1]. Thus in order to maintain the important interactions in organisms during evolution, crucial mutations in one interacting partner might be rescued by compensatory changes in the other partner. This concept is known as co-evolution, also known as correlated evolution or co-variation. Since there are not enough in vivo data available to test the co-evolution in RNA-protein interactions, we first use an in vitro dataset [5] of more than 200 RBPs to show that a significant correlation is observed between RBPs and their binding preferences. Then based on such correlation, we introduce a K-nearest neighbors algorithm to predict the sequence preference (represented by a PWM) for an RBP, using PWMs of the homologous neighbors as input. We evaluate the algorithm through a set of tests on the RBPs with known in vivo or in vitro binding data. We compare the KNN algorithm with (1) the alternative “inferred” approach in Ray et al.’s study [5] which used the closest neighbor’s preference (i.e. 1NN approach), (2) two state-of-the-art “direct” methods: DeepBind which represents the methods focusing on sequence preference [10], and RCK which represents the methods focusing on sequence-and-structure preference [17]. Our algorithm outperforms 1NN on all in vivo and in vitro datasets that have been tested, and even performs comparably on in vivo test sets in comparison to the “direct” methods DeepBind and RCK. In addition, we extend the RCK program to plug in our predicted PWMs as its sequence preference, in order to further incorporate structure preference. We show that the extended method performs comparably with DeepBind and RCK on in vitro test sets with smaller model and far less training time. Finally, we demonstrate the ability of our algorithm to predict binding preference for poorly studied RBPs, and we predict binding preferences for 1000 RBPs which do not have experimental data available.

## Methods

### Datasets

#### in vitro dataset

The first dataset was derived from a previously published RNAcompete study conducted by Ray et al. [5]. This study published results of 244 in vitro RNAcompete experiments for 207 RBPs from 24 eukaryotes. For each experiment, the study measured the RBP binding intensity for approximately 240,000 RNA probe sequences. Position frequency matrices were derived using top 10 probes for each experiment [5]. Many previous methods including 1NN, DeepBind, RCK/RNAcontext were all trained and tested on this dataset. We used the position frequency matrices in this dataset to form our training set, and the probes to form our in vitro testing set.

We performed several pre-processing steps on this dataset. We first filtered out proteins which contain more than one type of RNA-binding domains or protein families with too few members. We also removed the experiments with customized protein constructs to retain only the proteins with full-length (FL) or RNA-binding region (RBR, core binding region containing all RNA-binding domains in a protein), because Ray et al. cloned RBPs in different types of constructs [5]. In addition, for each RNAcompete experiment, probes with intensities above the 99.95th percentile were considered outliers and were clamped to the value of the 99.95th percentile as suggested in the studies of DeepBind and RCK [10, 17]. These steps made sure that we focus on the evolution of one protein family at a time and measure at both the FL sequence and the RBR levels. As a result, 200 out of the original 244 experiments remained after the pre-processing, which corresponds to the two largest RBP families known, the RNA Recognition Motif (RRM) family (177 in total: 126 RBR and 51 FL) and the K-homology (KH) family (23 in total: 15 RBR and 8 FL). We call this dataset the InVitro dataset for convenience. It covers RBPs from 24 diverse eukaryotes including animal, fungi, plant, and protist groups. The top three species with the most entries are human (74), Drosophila (56), C. elegans (10), *etc.* The detailed composition is listed in the Additional file 1. A summary of the InVitro dataset is shown in Table 2.

**Table 2:** Summary of datasets used in this study

Name	#	Source	Type	Species composition
InVitro	200	[5]	in vitro RNAcompete	24 different eukaryotes
InVivoRay	32	[5]	in vivo CLIP and RIP	human
InVivoAURA	9	[24]	in vivo CLIP	human

#### in vivo dataset

In addition, as shown in Table 2, we used two in vivo datasets to test the performance of in vitro derived binding preferences. The first one was the overlap of the in vivo dataset curated by Ray et al. [5] from different literatures with our InVitro dataset. It has 13 CLIP/RIP experiments corresponding to 14 RNAcompete proteins, which result in 32 RNAcompete-CLIP/RIP combinations. Each CLIP/RIP experiment here contains target RNA sequences with binary labels (i.e. “bound” or “unbound”), and has balanced samples for each label [5]. We call this dataset the InVivoRay dataset. All the corresponding RBPs in the InVivoRay dataset are from human, and most belong to the RRM family except one from the KH family. The detailed composition is listed in the Additional file 1. The second in vivo dataset was the overlap of the in vivo dataset derived by Cirillo et al. [24] from the AURA [25] database with our InVitro dataset. RNAs here are all long non-coding RNAs (lncRNAs). We got 6 overlapped combinations (out of 6 RNAcompete experiments and 2 CLIP experiments) with our InVitro dataset. Moreover, there are 3 additional CLIP experiments in this dataset that involve RBPs with no RNAcompete data, which provides a good case study to test the ability of our algorithm to infer binding preferences for the poorly studied RBPs. We call this dataset the InVivoAURA dataset. Furthermore, all the corresponding RBPs in the InVivoAURA dataset are from human, and most belong to the RRM family except one from the KH family.

### RBP binding preference model

#### Sequence preference

In this study, we used PWMs as our sequence preference representations. A PWM is a 4 (one for each nucleotide) by k (one for each position in a motif) matrix of base compositions (probabilities), which assumes position independence. Despite the fact that there are more advanced representations of binding preference which have weaker assumptions and capture more spatial relation [10, 16, 17], PWM has been the most commonly used representation, especially when integrating different models from various sources [19, 26]. We collected the position frequency matrices from the InVitro dataset, and converted them to PWMs with identical length (7) [22] (more details in Additional file 2: Supplementary Note). Then, for an RBP *x* of interest, we infer its PWM from its homologous PWMs using the KNN algorithm introduced below, without looking at *x*’s binding data like probe sequences, intensity values, *etc.*

Here we present our KNN based algorithm for sequence preference prediction. Suppose we are interested in a poorly characterized RBP *x* which only has its amino acid sequence available, eg. a novel protein that is newly discovered to be associated with certain disease. If we can find some of *x*’s homologous RBPs (either orthologs or paralogs, denoted by set *H*) that have known PWMs and map *x* to these RBPs by sequence identity, then we can predict the PWM of *x* with a non-parametric method similar to the K-nearest neighbors regression:

1. Compute a pairwise similarity *w*_*i*_ between RBP *x* and each RBP *h*_*i*_ in *H*, based on the sequence identity.
2. Sort *h*_*i*_ in descending order in terms of *w*_*i*_.
3. Find a *K* value which denotes the number of the nearest neighbors.
4. For the *K* nearest RBPs *h*^1^,..,*h*^*K*^ with similarities *w*^1^,..,*w*^*K*^ and PWMs *PWM*^1^,..,*PWM*^*K*^, predict *x*’s PWM with each cell (*i,j*) in *PWM*^*x*^ a weighted average:
  1 $$ PWM^{x}(i,j) = \frac{\sum_{p=1}^{K} w^{p}PWM^{p}(i,j)}{\sum_{p=1}^{K} w^{p}}  $$

Intuitively, our KNN algorithm assumes the RBPs and their binding motifs co-evolved perfectly, and infers the probability in each cell of the new PWM as a weighted average with weights equal to the similarities of protein sequences. The algorithm computes the sequence similarities using ClustalW [27]. Like the typical KNN algorithm, the proposed algorithm goes over different *K* values to find the optimal *K* (*optK*) for each RBP by cross validation. In this case, the different *K* values indicate different evolutionary distances between RBPs. Note the *K* (upper case) here denotes the number of neighbors and has nothing to do with the k (lowercase) in k-mer. In addition, to be consistent with the previous RNAcompete papers [4, 5], we used a similar approach as theirs to assign a score to an RNA sequence using a PWM. The predicted PWM with the length k (k was fixed to 7 in our case) assigns a score for any k-mer RNA sequence by taking the product of the PWM entries corresponding to each base in the k-mer. For an RNA sequence *s* with length |*s*|>*k*, the proposed algorithm scans *s* using the PWM to compute a RBP-binding score *y* for the entire sequence:

2 $$ \begin{aligned} y&= \frac{1}{|s|}\sum_{t=0}^{|s|-k} f\left(\prod_{l=t+1}^{t+k} PWM(index(s_{l}), l-t)\right), \text{where}~f(a)\\&= \left\{\begin{array}{cc} arcsinh(a), \quad a> 1\\ 0, \quad a\leq 1 \end{array}\right. \end{aligned}  $$

*index*(*s*_*l*_) returns the PWM’s row index for base *s*_*l*_. The use of *f*(*a*) guarantees that only k-mers with high scores are retained. This *y* score is used as our prediction for the binding intensity of an RNA probe.

#### Sequence-and-structure preference

Since the RNA structure is known to play a significant role in RNA-protein interactions [2, 28, 29] and more experimentally measured RNA structure data may be available in the future [7], we provide the flexibility of incorporating structure information with our predicted PWM. We chose to extend the recently published RCK program [17] which can infer both the sequence and the structure preferences using a k-mer based model. There are several reasons for choosing RCK: (1) it has a sequence-and-structure model with clear interpretation of each part; (2) it is suitable to plug in our PWM; (3) it was reported to have superior performance among others [17]. We modified the RCK’s sequence model so that it can take our PWM as input and use the parameters derived from our PWM instead of learning from sequence data. We then trained a joint model with the structure preference incorporated. In spite of the fact that our PWM was inferred without looking at the target RBP’s binding data, the rest of the model parameters were directly trained on the RBP’s RNAcompete probe data. Thus, this method is still a “direct” method. In addition, the RNA structure distribution was predicted computationally by a variant of RNAplfold [9, 15]. For simplicity, we call our modified RCK version KNN-RCK.

For each RBP *x*, KNN-RCK fits a model on *x*’s RNAcompete experiment data which consists of a set of probes and their binding intensities to *x*. Here an RNAcompete probe with length |*s*| is encoded as a vector *s* of nucleotides and a vector *p* of structural probabilities. We left the other parts of the RCK model untouched and focused on the sequence preference part *F*^*seq*^(·) which is a logistic function that estimates the probability of a given k-mer subsequence being bound by *x*:

3 $$ F^{seq}\left(s_{t+1:t+k}, \Theta\right)= \left(1+exp\left(-b-\phi_{s_{t+1:t+k}}\right)\right)^{-1}  $$

where *s*_*t*+1:*t*+*k*_ is the k-mer subsequence starting at *t*+1 on *s*, $\phi _{s_{t+1:t+k}}$ is the score parameter for this k-mer, and *b* is simply a bias term. *b*,*ϕ*∈*Θ*. For a given *k*, RCK assumes position dependence, and has a score parameter for each possible k-mer. Thus, *ϕ* has 4^*k*^ parameters. For instance, if *k*=5, *ϕ* would be a vector of parameters for all 5-mers: like *ϕ*_*AAAAA*_=0.03, *ϕ*_*CAAAA*_=1.20, *ϕ*_*GAAAA*_=−2.11, *etc.* In KNN-RCK, we have a PWM with length *k*, which assumes position independence and thus has 4×*k* parameters instead of 4^*k*^. In order to convert the PWM to *ϕ*, we used the PWM to score each possible k-mer *m* by simply multiplying the relevant probabilities at each position:

4 $$ \phi_{m}= \prod_{l=1}^{k} PWM(index(m_{l}), l)  $$

where *index*(*m*_*l*_) returns the PWM’s row index for base *m*_*l*_. When training KNN-RCK, we assigned these scores to *ϕ* at the initialization stage, and removed *ϕ* from parameter optimization. The rest parameters were still optimized the same way as in RCK.

### Assessing co-evolution in RNA-protein interaction

To test if the evolutions of the RBPs and their binding sequence preferences are correlated, we used a similar approach as in our previous study for measuring co-evolution between the transcription factors and their binding sites [22]. This approach was derived from the “mirror tree” method originally used in protein-protein co-evolution [30]. In brief, to assess the correlation, we derived a pairwise sequence similarity matrix for proteins and a pairwise similarity matrix for PWMs, then we computed a Pearson’s correlation coefficient (PCC) between these two matrices as the measure of co-evolution [30]. Each PWM represented a set of RNA targets for an RBP. Since this approach is basically the same as our previous study and is not the focus here, the details are described in the Additional file 2: Supplementary Note.

### Evaluating prediction performance

We evaluated our predicted binding preference through a series of tests on the in vitro and in vivo datasets. Firstly, for the in vitro testing, we performed leave-one-out validation for all the proteins in our InVitro dataset. Each time for an experiment with the target RBP *x*, we pretended not to have the binding data for *x* and trained a PWM with our KNN algorithm using only the homologous proteins’ PWMs. In the original study of Ray et al. [5], the probes in the InVitro dataset were split into two sets A and B which have similar sizes and k-mer coverages. We trained our KNN on the homologous PWMs derived from set A, and selected the optimal K value using 2-fold cross-validation on the probes (and intensities) from A. Then we tested on the probes (and intensities) from B. Since the probe intensities are continuous, the performance was evaluated by PCC between the predicted and the real intensities. In the DeepBind and the RCK papers, these two methods were also trained on set A and tested on B using PCC, except they were directly trained on the target RBP’s probe data [10, 17]. So we just used their published performance results. In addition, we also trained our KNN-RCK algorithm the same way as RCK did in its paper to incorporate the structure preference.

The more important evaluation is the in vivo testing. All the methods were trained on the complete InVitro dataset (set A+B) then tested on the two in vivo datasets, respectively. Since RNA sequences in the InVivoRay and InVivoAURA datasets were labeled as bound and unbound, the performance was evaluated by the area under the receiver operating characteristic curve (AUC). For the InVivoRay dataset, there were 6 InVitro RBPs with each corresponding to multiple in vivo test sets. The previous Ray et al. study selected the test set with the best performance for each RBP [5]. Here, we simply took the average performance of all the test sets for each case, and obtained 16 entries from the total 32. To be consistent with the RCK paper [17], we used 2-fold cross-validation to determine RCK’s hyper-parameter width (4–7) on the entire InVitro dataset, and then tested on the InVivoRay dataset with optimal width. The DeepBind study did the same evaluation procedure as ours to test on the InVivoRay dataset [10]. So we again used the performance results in the DeepBind paper. For the InVivoAURA dataset, we tested DeepBind using its published pre-trained preference models since its training took too long. We ran DeepBind with ‘-average’ option turned on to be consistent with the DeepBind paper [10]. For the RBPs in the InVivoAURA that did not overlap with the InVitro dataset (i.e. novel RBPs), DeepBind and RCK could not deal with such cases. We used the preference model of the nearest neighbor available for each novel RBP instead, i.e. the same idea as 1NN.

## Results

### Correlation between the RBPs and their RNA targets

First we tested the co-evolution in RNA-protein interactions. Since the RRM and the KH families are the two families composing our InVitro dataset, we focused on them to assess such correlation. As described earlier, the protein constructs in the InVitro dataset were either FL sequences or RBR fragments. Hence, we separated the cases of RRM-FL, RRM-RBR, KH-FL, and KH-RBR for the analysis.

As shown in Table 3, the values under the PCC column stand for the measured co-evolutions. To assess the significance of the PCC value, we used a nonparametric rank test and a parametric test as suggested in Yang et al.’s study [22] (Additional file 2: Supplementary Note). In both tests, the KH-FL and the RRM-FL sets showed significant correlation with *p*-value < 0.05 and *p*-value < 0.01, respectively. In the nonparametric test, the KH-RBR and the RRM-RBR sets showed significant correlations with *p*-value < 0.05 (*). The parametric test is more stringent: the KH-RBR set had a *p*-value = 0.158 and the RRM-RBR set had a *p*-value = 0.057, which was close to 0.05 significant level. The fact that the FL level displayed more significant correlations than the RBR level may indicate that: although the binding domain is the most relevant factor to the RNA contacting, the rest of the protein sequence might have a long range effect on the RNA recognition and binding, which would provide additional evolutionary information. In addition, since each of the RRM-FL, the RRM-RBR, the KH-FL, and the KH-RBR sets contains proteins from multiple species, we also controlled the effects of speciation and confirmed the observed correlations were not due to speciation (Additional file 2: Supplementary Note and Figure S1). In summary, we observed strong correlations between the RBPs and their PWMs in our InVitro dataset.

**Table 3:** Co-evolution between RBPs and their RNA targets

RBP family	Construct^1^	# of members	PCC
KH	FL	8	0.760*
RRM	FL	51	0.419**
KH	RBR	15	0.367(*)
RRM	RBR	126	0.174(*)

^1^: Protein construct, FL stands for full length protein, and RBR stands for RNA-binding region. *: *p*-vale  < 0.05, ** *p*-value < 0.01 from both the parametric and nonparametric tests; (*): *p*-value < 0.05 from the nonparametric test

### Performance of our “inferred” method for preference prediction

In order to assess the ability of our method in predicting the binding preference for novel or poorly characterized RBPs, first we applied our KNN algorithm to the RBPs with known binding data and demonstrated the ability of our algorithm. We compared the performance of our method with 1NN, DeepBind and RCK on the InVitro and InVivoRay datasets.

#### Our method is more accurate than the alternative “inferred” method

**Fig. 1:**
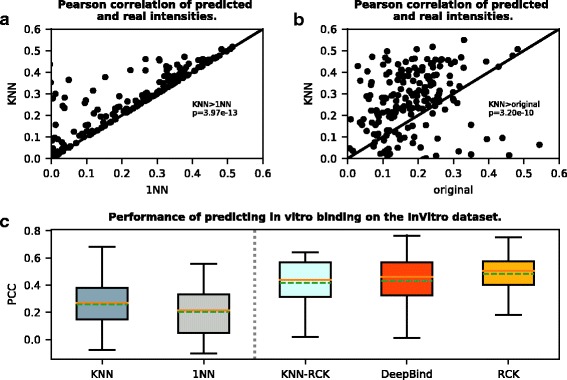
Performance in predicting in vitro binding on the InVitro dataset. For each RBP, all methods were trained and tested on the InVitro dataset. Performance was measured by PCC of the predicted and real RNAcompete probe intensities. **a** Scatter plot shows our KNN (with optimal K) predicted PWMs perform better than or as well as 1NN predicted PWMs in all RBPs in terms of the PCCs of predicted and true probe intensities. *p*-value is calculated by paired t-test. **b** Scatter plot shows our KNN predicted PWMs also outperform the left-out original PWMs derived by Ray et al. [5]. **c** Box plot of PCCs for different methods including KNN, 1NN [5], DeepBind [10], RCK [17], and KNN-RCK. The vertical dashed line separates boxes for methods requiring only the target RBP’s homologous binding information for training to the left, and methods requiring the target RBP’s explicit binding data for training to the right. In each box, the dashed green line denotes the mean, and the brown line denotes the median

To compare with the alternative “inferred” method 1NN, we evaluated the performances of our KNN with *K*=*optK* and *K*=1. The 1NN case corresponds to the method used in Ray et al.’s study [5]. We first gauged the in vitro performances on the 200 experiments in the InVitro dataset. As described in the Methods section, we did it in a leave-one-out fashion. As shown in Fig. 1 (a and c) and Table 4, KNN actually outperformed 1NN on every experiment, with an average PCC 0.257 as compared to 0.202 for 1NN. The *p*-value from a paired t-test between the KNN’s PCCs and the 1NN’s PCCs was around 10^−13^. In addition, we compared the performance of our KNN predicted PWMs with the left-out original PWMs derived by Ray et al. [5]. As shown in Fig. 1b, our KNN predicted PWMs performed much better (*p*-value around 10^−10^) even than the original left-out PWMs. This is encouraging because for each protein x in the dataset, its original PWM was derived directly from the RNAcompete probes, while its KNN inferred PWM was derived indirectly without the probe information but only using the homologous proteins’ original PWMs.

**Table 4:** Overview of different methods that were evaluated

Method	Training data	Model	Testing data		Performance		
			in vitro	in vivo	InVitro	InVivoRay	InVivoAURA
1NN [5]	PWM	PWM	p, i	t, l	0.202^1^	0.736^2^	0.682^2^
DeepBind [10]	p, i	CNN^3^	p, i	t, l	0.429	0.791	0.671
RCK [17]	p, s, i	k-mer	p, s, i	t, s, l	0.484	0.708	0.539
**KNN**	PWMs	PWM	p, i	t, l	0.257	0.818	0.714
**KNN-RCK**	PWM, p, s, i	customized k-mer	p, s, i	t, s, l	0.417	0.664	-

^1^: Pearson correlation averaged over all tested proteins. ^2^: AUC averaged over all tested proteins. ^3^: Convolutional neural network. In the second column, p: RNAcompete probe sequences. i: RNAcompete probe intensities. s: predicted structural distribution. t: CLIP/RIP binding transcript segment sequences. l: CLIP/RIP binary label for bound or unbound

**Fig. 2:**
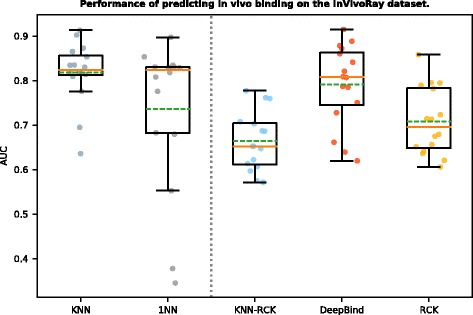
Performance in predicting in vivo binding on the InVivoRay dataset. For each RBP, all methods were trained on the InVitro dataset and tested on the InVivoRay dataset. Performance was measured by AUC of the predicted and real (CLIP/RIP) binary labels. The figure shows the box plot of AUCs for different methods including KNN, DeepBind [10], 1NN [5], RCK [17], and KNN-RCK. The vertical dashed line separates boxes for methods requiring only the target RBP’s homologous binding information for training to the left, and methods requiring the target RBP’s explicit binding data for training to the right. In each box, the dashed green line denotes the mean, and the brown line denotes the median

Moreover, since the in vitro performance was trained and tested on the same type of RNAcompete data, we then investigated whether the PWMs trained on the RNAcomplete data generalized well on the in vivo data, which is a more important task for the RNAprotein interaction study. As shown in Table 4 and Fig. 2, when evaluated on the 32 in vivo entries in the InVivoRay dataset, the PWMs predicted by KNN achieved an average AUC 0.818, comparing to 1NN AUC 0.736. The corresponding *p*-value was < 0.05 from a paired t-test. Thus, in general, we observed a strong improvement of using our KNN algorithm as opposed to the 1NN. This also confirmed that the co-evolution detected from the in vitro data also exists in vivo.

#### Our method is comparable to the state-of-the-art “direct” methods

Next we compared the performance of our “inferred” method to the “direct” methods DeepBind and RCK. For the in vitro binding prediction, as we can find in Table 4 and Fig. 1c, the performance of DeepBind (average PCC = 0.429) and RCK (average PCC = 0.484) were much better than our KNN (average PCC = 0.257). However, both the “direct” methods DeepBind and RCK were trained to predict the RNAcompete probe intensity, and were directly optimized to minimize the difference between the predicted and the real probe intensities as the objective function. Our KNN was not trained to directly predict the intensity, which was obviously disadvantageous when using the intensity as evaluation criteria.

To make the comparison fairer, we evaluated our KNN-RCK which was also trained to directly predict the RNAcompete probe intensity. As a result, we got an average PCC = 0.417 which was much closer to DeepBind (no statistically significant difference) and RCK (still significantly stronger), with much less training time. When trained on one RNAcompete experiment (using set A only) on the same machine, KNN-RCK took < 1 hr (53min on average over a subset of 14 experiments); RCK took 3–4 hrs (220min on average), both with the hyper-parameter width = 7. The time was evaluated on a single Intel Xeon E5-2690 (2.90GHz) CPU with 8GB RAM. DeepBind is not comparable regarding the time since it needs GPU for training, which is much more computationally intensive. In our empirical test, DeepBind did not finish training in 24 hrs. The time was based on a single NVIDIA Tesla M2070s GPU (5.5 GB memory) of a 12-CPU Intel Xeon E5694 (2.53GHz) machine with 23GB RAM. It is also worth noting that the sizes of our models (i.e. number of parameters) are much smaller than the models fitted by DeepBind and RCK (Table 4): our KNN simply has a PWM as its model which contains only 4×*k* (k was fixed to 7) parameters; DeepBind typically has thousands of parameters (depending on the settings of its many hyper-parameters); RCK has about 4^*k*^ sequence parameters (k is within 3-7, determined through cross validation), and 4^*k*^×*c* (c is the number of structural contexts, and by default equals to 5) structure parameters (plus a few regression terms); KNN-RCK is smaller than RCK since using PWM computed scores as the sequence model, and has about 4^*k*^×*c*+4×*k* parameters.

Moreover, we also compared the in vivo binding prediction. As shown in Table 4 and Fig. 2, KNN, RCK and DeepBind showed comparable performance. Our KNN method had the highest AUC (0.818) on average, which was significantly better than RCK (AUC 0.708, *p*-value around 10^−4^), and also slightly better than DeepBind (AUC 0.791). This may reflect that the complicated models like DeepBind and RCK have a higher variance in prediction and tend to overfit to the training data compared to the simple models like KNN. We also compared the performance of our KNN predicted PWMs with the original PWMs derived by Ray et al. [5]. On the InVivoRay dataset, the original PWMs got an average AUC = 0.785, and was again worse than KNN (0.818, *p*-value = 0.019). Besides, KNN-RCK (AUC 0.664) was significantly worse than KNN (AUC 0.818) in this test (*p*-value around 10^−6^). The reasons for the sequence-and-structure preference models like RCK and KNN-RCK not performing as well as the sequence preference models like DeepBind and KNN may be that: (1) As the training data, the RNAcompete probes were designed to be short (30-41 nt) and have weak secondary structures. While as the testing data, the RNA segments from CLIP/RIP experiments were usually much longer (many > 1000 nt) and tended to form much more structures (also harder for computational structure prediction) [5]. (2) The RNA sequences in InVivoRay were only transcript segments which did not include flanking regions so that the predicted structures might be inaccurate. These also reflected the limitation of RCK (and KNN-RCK) which requires not only the binding sequence data but also the accurate structure annotation to be available to make a decent prediction. In summary, the results here showed that although our KNN method requires only homologous proteins’ PWMs as input, its performance was comparable to the much more complicated state-of-the-art methods when testing on in vivo binding data.

In addition, we further utilized KNN-RCK’s binding preference model to assess the relative importance of the sequence or the structure feature alone, regarding binding prediction. Note that although DeepBind represents the sequence-based methods and RCK represents the sequence-and-structure methods, we cannot simply compare the performance of DeepBind with RCK to assess the relative importance since their models and training algorithms are very different. So we did the assessment under KNN-RCK’s unitary framework to control the irrelevant effects. The results were presented and discussed in the Additional file 2: Supplementary Note and Figure S2.

### Case study: our method infers PWM for novel proteins

**Fig. 3:**
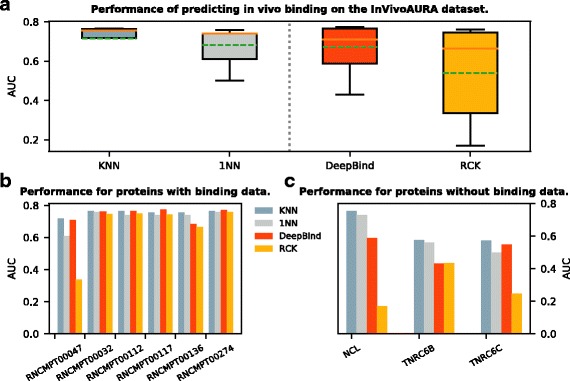
Performance in predicting in vivo binding on the InVivoAURA dataset. For each RBP, all methods were trained on the InVitro dataset and tested on the InVivoAURA dataset. Performance was measured by AUC of the predicted and real (CLIP) binary labels. **a** Box plot of AUCs for different methods including KNN, DeepBind [10], 1NN [5], and RCK [17]. The vertical dashed line separates boxes for methods requiring only the target RBP’s homologous binding information for training to the left, and methods requiring the target RBP’s explicit binding data for training to the right. In each box, the dashed green line denotes the mean, and the brown line denotes the median. **b** Bar plot of AUCs for RBPs (named by model IDs) with explicit binding data available for training. **c** Bar plot of AUCs for RBPs with no binding data but only homologous binding information available for training. **b** and **c** are the performances breakdown for each group of RBPs (well studied, poorly studied) from **a**

Here we used the InVivoAURA dataset as a case study to further demonstrate the ability of our KNN algorithm to predict the binding preference for the novel or poorly studied RBPs. As introduced in the Methods section, this dataset contains 9 sets of lncRNA-protein interactions (on average > 1000 nt long, entire 3’UTR/5’UTR) with 3 out of them having no RNAcompete information. As shown in Table 4 and Fig. 3a, overall KNN (average AUC = 0.714) performed the best among all four methods (1NN:0.682, DeepBind:0.671, RCK:0.539) and was significantly better than 1NN (*p*-value = 0.021) and RCK (*p*-value = 0.035). To elaborate, we first looked at the two RBPs ELAVL1 and QKI which have known RNAcompete binding data (ELAVL1 corresponds to RNCMPT00032, RNCMPT00112, RNCMPT00117, RNCMPT00136, RNCMPT00274; QKI corresponds to RNCMPT00047) to train. As shown in Fig. 3b, for ELAVL1, all four programs KNN, 1NN, DeepBind and RCK gave similar AUCs with KNN slightly better than the rest (KNN still significantly better than 1NN with *p*-value = 0.014); then for QKI, KNN also had the highest AUC (0.718) with DeepBind very close to it (0.709). Next, for the remaining three RBPs (NCL, TNRC6B, TNRC6C), there was no RNAcomepte data available, which served as the case for the poorly studied proteins. Since all three RBPs can be mapped to the RRM family (FL) based on the protein sequence identity, we could use our KNN method as before to predict the PWMs for them. Here we predicted with a fixed K = 7 (average of opt-K values over all experiments from training) to find the proper homologous proteins’ PWMs in the InVitro dataset. For DeepBind and RCK, since they did not have the corresponding InVitro data to train, we used the model of the nearest neighbor from the InVitro dataset for each of the three RBPs (NCL:RNCMPT00009, TNRC6B:RNCMPT00094, TNRC6C:RNCMPT00179). Our KNN method performed the best in all three cases (Fig. 3c). Especially, it outperformed DeepBind and RCK by a large margin (except for DeepBind in TNRC6C), which suggested the capability and necessity of our KNN method for the poorly studied proteins.

Finally, after demonstrating the capability of our KNN method, we made inference of PWMs for 1000 poorly studied RBPs selected from cisBP-RNA database [19]. These RBPs contain either KH or RRM RNA-binding domain, from a diverse range of eukaryotes. They were categorized as “inferred” in the “motif evidence” menu in the cisBP-RNA database, and were previously inferred for their binding preferences by 1NN method [5]. We predicted the PWMs for these proteins by KNN and expected the new PWMs would be more accurate than the previous 1NN inferred ones. The PWMs are available on our website.

## Discussion

The main contribution of this study is to predict the binding preferences for poorly characterized RBPs by utilizing co-evolution. It would be ideal if we could directly determine an RBP’s preference from its experimental binding data. However, such data is currently missing for most proteins. So here we explore how to indirectly infer the preferences for poorly studied RBPs in the absence of their binding data. We conducted a co-evolutionary analysis on an in vitro RNAcompete dataset which is the largest RNA-protein binding dataset by far and is known to correlate well with the in vivo data [4, 5]. Based on the existence of such co-evolution, we proposed a KNN algorithm to integrate the binding preferences of the homologs into the binding preference prediction. We then benchmarked its performance on the in vivo as well as the in vitro binding data available, and compared it with several representative “direct” and “inferred” methods. The performance was especially well on the in vivo data. By taking an independent lncRNA dataset as a case study, we further demonstrated how to use the algorithm for poorly studied RBPs which do not have binding data in practice.

To predict the binding preference for a poorly studied RBP, our method requires the presence of a set of homologs with known preferences. Although the existing datasets, such as the InVitro RNAcompete dataset, provide good sources of homologous proteins with PWMs, the homologous data is still very limited for most RBPs. So currently, for a query RBP, our method uses the InVitro dataset as the source and combines information of both orthologs and paralogs from it to make preference predictions. However, the idea underlying our KNN method is that the homologous RBPs highly co-evolve with their binding motifs subject to the evolutionary selection. It was derived from the famous “mirror tree” approach to measure protein-protein co-evolution [30], which uses the orthologs only. We relaxed this requirement here due to the limited availability of data. If more orthologs data become available in the future, our method will be restricted to use orthologs only.

**Fig. 4:**
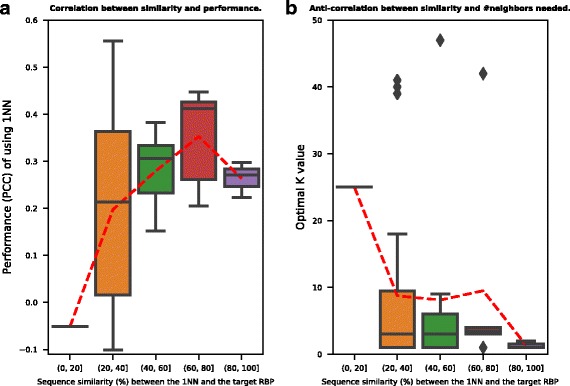
Analyses of the number of homologous RBPs and their sequence similarities to the target RBP for the KNN algorithm. The figure is based on the RRM-FL set from the InVitro dataset. **a** Box plot of the preference prediction performances for five different sequence similarity bins. The x-axis shows the similarity between the target RBP and the nearest neighbor (1NN). The y-axis shows the in vitro performance (PCCs) of using 1NN for preference prediction. The red dashed line connects the mean value of each bin. A significant correlation (0.3, *p*-value < 0.05) was observed between the PCC and the sequence similarity values. **b** Box plot of the number of neighbors needed for five different sequence similarity bins. The x-axis is the same as that in **a**. The y-axis denotes the optimal number of neighbors (optK) to use in the KNN algorithm. The optK value was determined through cross validation. A negative correlation (-0.17) was observed between the optK and the sequence similarity values

It is desirable to understand how the KNN method works in terms of the number of neighbors (i.e. homologs). Here we provide some intuition. As described in the Method section, the optimal number of neighbors to use in the algorithm is determined by cross validation. The question is why some RBPs have small optK values (eg. optK = 1) while others need much larger values (eg. optK = 30). We make the general observation that the closer the neighbors to the target RBP, the smaller the number of neighbors needed to make the prediction. To illustrate this observation, we use the RRM-FL set from in vitro testing as an example. In Fig. 4a, the x-axis shows the global sequence similarity between the target RBP and *the* nearest neighbor (1NN). In the RRM-FL set, there are 51 RBPs. We sorted the 1NN similarity values and put them into five bins. The y-axis shows the performance (PCC) of using 1NN for preference prediction. The right-hand-side bins corresponding to more similar 1NN neighbors show better performance in general. And when the 1NN similarity is low, the prediction performance by using 1NN only is poor. The red dashed line connects the mean value of each bin in Fig. 4a. There is a positive correlation (0.30) between the 1NN similarity and the prediction performance (*p*-value < 0.05). To generalize from using 1NN only for prediction to the proposed algorithm of using optK for prediction, Fig. 4b shows a general anti-correlation (−0.17) between the optimal number of neighbors needed and the similarity to the nearest neighbor. While the x-axis in Fig. 4b is the same as that in Fig. 4a, the y-axis shows the optimal number of neighbors (optK). The right-hand-side bins generally require smaller numbers of neighbors for prediction. And when the 1NN similarity is low, a larger number of neighbors are needed.

Although an RNA molecule could fold onto itself to form functionally important structures, we focus on the sequence preference of RNA in this study. One main reason is that there are too few experimentally determined structure data available at this moment. Moreover, the computationally predicted RNA structure tends to be less accurate for longer sequence [31]. Another reason is that many RBPs are sequence-specific and essentially bind to single-stranded RNAs [2], such as RRM and KH family in this study. However, as we expect that more structure data will be available in the future, we provide the KNN-RCK algorithm to recruit structure features as well. We can assess the importance of structure or sequence information alone for binding preference prediction using KNN-RCK.

In general, predicting RBPs’ binding preferences is challenging, especially for the RBPs with no available experimental binding data. The simple KNN method we introduced here exhibits considerable potential for this task, and can be further extended in several directions. Firstly, one limitation of the current version is that the widths of all the PWMs are fixed to be the same. It may be interesting to make the width of the PWMs an variable that can be tuned for different RBPs. Furthermore, when predicting preferences for novel RBPs with no binding data, the optK value for KNN is currently fixed to an empirical value for different RBPs. It would be interesting to explore more about the co-evolutionary relationship to see if we could customize the optK for each novel RBP. Finally, our KNN algorithm is ready to be used in other scenarios such as the transcription factor binding preference detection. The binding preference model in our KNN does not have to be PWMs, and could be replaced by other models (like the k-mer model in RCK, or the convolution function in DeepBind) instead. In general, this study provides a flexible framework to investigate the dynamics of the nucleotide-protein interactions in cell through evolution, and supplies a practical solution that is easy to use for the research community.

## Conclusions

In this study we presented a novel method to predict the binding preference for the poorly studied RBPs. First we examined the co-evolution in the RNA-protein interaction using data available in vitro. Then we described a KNN based RBP sequence preference prediction algorithm utilizing such correlation. We evaluated our predicted preferences on several datasets both in vitro and in vivo. Moreover, we explored how to use our KNN based method to infer sequence preferences for the unknown or poorly studied RBPs. This study is the first to explicitly explore the co-evolution to predict the RBP binding preference. It has the potential to reveal the existence of the complicated interaction codes between RNAs and proteins, and study the vast majority of the RBPs that are pending to be characterized.

## Additional files

Additional file 1 Supplementary Data. The excel document contains the Supplementary Data about the performances of different methods on InVitro, InVivoRay and InVivoAURA datasets, as well as the composition of each datasets (including species and RNA-binding domain information). (XLSX 32 kb)

Additional file 2 Supplementary File. The PDF document contains texts for the Supplementary Note, and the Supplementary Figures S1 to S2. **Figure S1** shows the PCCs of KH RBP, RRM PWM pairs for 1000 randomly shuffled sets. **Figure S2** shows the comparison of the full sequence-and-structure, structure alone, and sequence alone models in KNN-RCK, in terms of their performances in predicting (A) in vitro binding on the InVitro dataset (B) and in vivo binding on the InVivoRay dataset. (PDF 217 kb)

## Abbreviations

AUC: Area under the receiver operating characteristic curve
CLIP: Crosslinking immunoprecipitation
FL: full-length
KH: K-homology
KNN: K nearest neighbors
lncRNA: long non-coding RNA
PCC: Pearson correlation coeffcient
PWM: Position weight matrix
RBP: RNA-binding protein
RBR: RNA-binding region
RIP: RNA immunoprecipitation
RRM: RNA recognition motif

## References

[1] Jankowsky E, Harris ME (2015). Specificity and nonspecificity in rna-protein interactions. Nat Rev Mol Cell Biol.

[2] Li X, Kazan H, Lipshitz HD, Morris QD (2014). Finding the target sites of rna-binding proteins. Wiley Interdiscip Rev RNA.

[3] Wang T, Xiao G, Chu Y, Zhang MQ, Corey DR, Xie Y. Design and bioinformatics analysis of genome-wide clip experiments. Nucleic Acids Res. 2015. 10.1093/nar/gkv439. 10.1093/nar/gkv439 PMC4477666 25958398

[4] Ray D, Kazan H, Chan ET, Castillo LP, Chaudhry S, Talukder S, Blencowe BJ, Morris Q, Hughes TR (2010). Rapid and systematic analysis of the rna recognition specificities of rna-binding proteins. Nature Biotech.

[5] Ray D, Kazan H, Cook KB, Weirauch MT, Najafabadi HS, Li X, Gueroussov S, Albu M, Zheng H, Yang A, Na H, Irimia M, Matzat LH, Dale RK, Smith SA, Yarosh CA, Kelly SM, Nabet B, Mecenas D, Li W, Laishram RS, Qiao M, Lipshitz HD, Piano F, Corbett AH, Carstens RP, Frey BJ, Anderson RA, Lynch KW, Penalva LOF, Lei EP, Fraser AG, Blencowe BJ, Morris QD, Hughes TR (2013). A compendium of rna-binding motifs for decoding gene regulation. Nature.

[6] König J, Zarnack K, Luscombe NM, Ule J (2012). Protein–rna interactions: new genomic technologies and perspectives. Nat Rev Genet.

[7] Spitale RC, Flynn RA, Zhang QC, Crisalli P, Lee B, Jung J-W, Kuchelmeister HY, Batista PJ, Torre EA, Kool ET, Chang HY (2015). Structural imprints in vivo decode rna regulatory mechanisms. Nature.

[8] Janssen S, Giegerich R (2015). The rna shapes studio. Bioinformatics.

[9] Bernhart SH, Hofacker IL, Stadler PF (2006). Local rna base pairing probabilities in large sequences. Bioinformatics.

[10] Alipanahi B, Delong A, Weirauch MT, Frey BJ (2015). Predicting the sequence specificities of dna- and rna-binding proteins by deep learning. Nature Biotech.

[11] Foat BC, Houshmandi SS, Olivas WM, Bussemaker HJ (2005). Profiling condition-specific, genome-wide regulation of mrna stability in yeast. Proc Natl Acad Sci U S A.

[12] Pelossof R, Singh I, Yang JL, Weirauch MT, Hughes TR, Leslie CS (2015). Affinity regression predicts the recognition code of nucleic acid–binding proteins. Nat Biotechnol.

[13] Hiller M, Pudimat R, Busch A, Backofen R (2006). Using rna secondary structures to guide sequence motif finding towards single-stranded regions. Nucleic Acids Res.

[14] Li X, Quon G, Lipshitz HD, Morris Q (2010). Predicting in vivo binding sites of rna-binding proteins using mrna secondary structure. RNA.

[15] Kazan H, Ray D, Chan ET, Hughes TR, Morris Q (2010). Rnacontext: A new method for learning the sequence and structure binding preferences of rna-binding proteins. PLoS Comput Biol.

[16] Maticzka D, Lange SJ, Costa F, Backofen R (2014). Graphprot: modeling binding preferences of rna-binding proteins. Genome Biol.

[17] Orenstein Y, Wang Y, Berger B (2016). Rck: accurate and efficient inference of sequence- and structure-based protein–rna binding models from rnacompete data. Bioinformatics.

[18] Stormo GD (2000). Dna binding sites: representation and discovery. Bioinformatics.

[19] CISBP-RNA Database: Catalog of Inferred Sequence Binding Preferences of RNA Binding Proteins. http://cisbp-rna.ccbr.utoronto.ca. Accessed 20 June 2017.

[20] de Juan D, Pazos F, Valencia A (2013). Emerging methods in protein co-evolution. Nature Review Genetics.

[21] Weigt M, White RA, Szurmant H, Hoch JA, Hwa T (2009). Identification of direct residue contacts in protein–protein interaction by message passing. Proc Natl Acad Sci.

[22] Yang S, Yalamanchili HK, Li X, Yao K-M, Sham PC, Zhang MQ, Wang J (2011). Correlated evolution of transcription factors and their binding sites. Bioinformatics.

[23] Mahony S, Auron PE, Benos PV (2007). Inferring protein–dna dependencies using motif alignments and mutual information. Bioinformatics.

[24] Cirillo D, Blanco M, Armaos A, Buness A, Avner P, Guttman M, Cerase A, Tartaglia GG (2017). Quantitative predictions of protein interactions with long noncoding rnas. Nat Methods.

[25] Dassi E, Re A, Leo S, Tebaldi T, Pasini L, Peroni D, Quattrone A (2014). Aura 2. Translation.

[26] Cook KB, Kazan H, Zuberi K, Morris Q, Hughes TR (2011). Rbpdb: a database of rna-binding specificities. Nucleic Acids Res.

[27] Larkin MA, Blackshields G, Brown NP, Chenna R, McGettigan PA, McWilliam H, Valentin F, Wallace IM, Wilm A, Lopez R, Thompson JD, Gibson TJ, Higgins DG (2007). Clustal w and clustal x version 2.0. Bioinformatics.

[28] Re A, Joshi T, Kulberkyte E, Morris Q, Workman CT (2014). RNA-Protein Interactions: An Overview, vol. 1097.

[29] Dieterich C, Stadler PF (2012). Computational biology of rna interactions. Wiley Interdiscip Rev RNA.

[30] Pazos F, Valencia A (2001). Similarity of phylogenetic trees as indicator of protein–protein interaction. Protein Eng.

[31] Hofacker I, Gorodkin J, Ruzzo WL (2014). Energy-directed rna structure prediction. RNA Sequence, Structure, and Function: Computational and Bioinformatic Methods, vol. 1097.

