# Supplementary material for: Inferring RNA sequence preferences for poorly studied RNA-binding proteins based on co-evolution
Source: BMC Bioinformatics. 2018 Mar 12;19:96. doi: 10.1186/s12859-018-2091-8 (PMC5848454; doi:10.1186/s12859-018-2091-8)
Supplement: Supplementary file 2 — Supplementary File. The PDF document contains texts for the Supplementary Note, and the Supplementary Figures S1 to S2. Figure S1 shows the PCCs of KH RBP, RRM PWM pairs for 1000 randomly shuffled sets. Figure S2 shows the comparison of the full sequence-and-structure, structure alone, and sequence alone models in KNN-RCK, in terms of their performances in predicting (A) in vitro binding on the InVitro dataset (B) and in vivo binding on the InVivoRay dataset. (PDF 217 kb) [file 12859_2018_2091_MOESM2_ESM.pdf]

Supplementary Note for

Inferring RNA sequence preferences for poorly studied RNA-binding proteins based on co-evolution

Shu Yang, Junwen Wang and Raymond T. Ng

Contents

1 Converting position frequency matrices to PWMs 1

2 Measuring the co-evolution between RBPs and their binding sites 1

2.1 PWM-PWM pairwise similarity . . . 2

2.2 Assess the significance of co-evolution . . . 2

2.3 Control for the effects of speciation . . . 3

3 The importance of sequence or structure preference alone 3

1 Converting position frequency matrices to PWMs

The position frequency matrices from the InVitro dataset were converted to PWMs. For each matrix, we substituted all the zero entries with a small pseudo number. Then for all the entries, we divided each of them by a background distribution (the assumed background probability for each base is 0.25). In addition, the position frequency matrices have different lengths because of the experiment setting [1]. Since the majority of the matrices are 7 positions long and the rest matrices are either 8 or 9 positions long, when predicting the PWM of a target RBP by the KNN algorithm, we trimmed the longer matrices so that each PWM has the same length (i.e. 7 positions). Similar to the strategy we adopted in Yang *et al.*'s study [2], we trimmed the PWMs based on the information content, familial PWMs (constructed by STAMP program [3]), and manual adjustment.

2 Measuring the co-evolution between RBPs and their binding sites

The co-evolution measurement approach was used in our previous study for DNA-protein interaction (please refer to the article for details) [2], and was derived from the "mirror tree" method originally used in the protein-protein co-evolution by Pazos *et al.* [4]. The intuition is that we can build a multiple sequence alignment with a set of RBP sequences from the same family but different species (i.e. orthologs), then derive a phylogenetics tree. If each RBP has a corresponding RNA binding site and the RBP co-evolves perfectly with the RNA, then we could construct a phylogenetics tree which looks perfectly symmetrical (like a mirror) to the RBP tree from these RNA sequences. In reality, however, it is unlikely to observe exactly mirrored trees since the co-evolution is usually not perfect. Also, due to the limited data available at present, the proteins are not necessarily orthologs (could be paralogs or mix). As suggested in the Pazos *et al.*'s study [4], it is not necessary to construct the phylogenetics trees since the structure information of the phylogenetics tree is comprised in a similarity matrix. By constructing the similarity matrices, the "mirror tree" method is independent of any given tree-construction method. Therefore, instead of the phylogenetics trees, we constructed a pairwise similarity matrix based on the protein sequence identity for each protein family. We used ClustalW [5] to build a multiple-sequence alignment and computed pair-wise sequence

similarities. Correspondingly, we also constructed a pairwise similarity matrix for RNA motifs i.e. PWMs. We used several different metrics to compare the similarity of a pair of PWMs, which we will describe in the next paragraph. Finally, to quantify the co-evolution between RBPs and their binding motifs, we compared the RBP pairwise similarity matrix with the RNA pairwise similarity matrix directly by concatenating all the rows in each matrix into a vector and computing a Pearson's correlation coefficient, as suggested in the Pazos *et al.*'s study [4].

2.1 PWM-PWM pairwise similarity

The main principle to compare a pair of PWMs is as follows: assuming a PWM contains 4 rows (one for each base) and k columns (one for each position), to compare two matrices, we only need to compare the similarity values of individual column pairs and use them to compose the overall value on matrix level. To measure the similarity, we tried two different metrics. The first one is Pearson's correlation, again. PCC has been suggested as a standard way for DNA PWM comparison and has superior performance than other measures such as Euclidean distance or Chi-squares according to several studies [6–9]. We used PCC (different from co-evolution PCC) as the similarity measure to compare two PWMs by computing the correlation of each position (represented by column vector) pair of the two PWMs then averaging over all positions, as suggested in Yang *et al.* [2].

$$Corr(x,y) = \frac{\sum_{i=A,C,G}(x_i-\bar{x})(y_i-\bar{y})}{\sqrt{\sum_{i=A,C,G}(x_i-\bar{x})^2}\sqrt{\sum_{i=A,C,G}(y_i-\bar{y})^2}} \tag{S1}$$

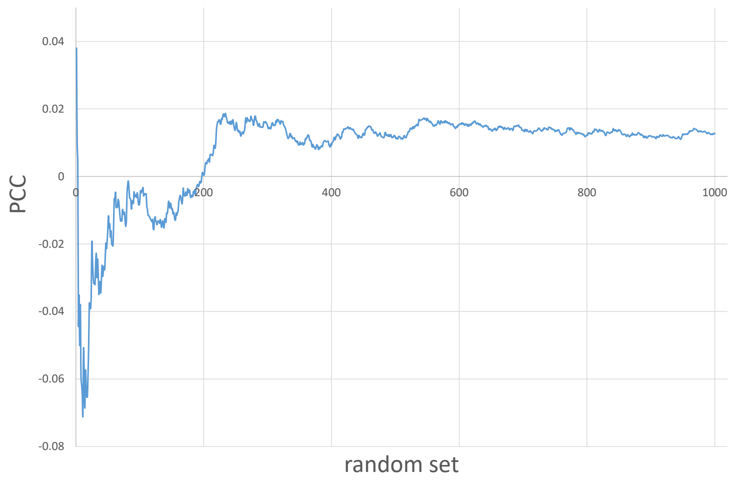

Figure S1: **PCCs for shuffled pairs.** The figure shows PCCs from 1000 randomly shuffled sets. For each set, a PCC is computed using random KH RBP, RRM PWM pairs from the same species at RBR level.

2.2 Assess the significance of co-evolution

As suggested in Yang *et al.*'s study [2], here we used both a nonparametric test and a parametric test to assess the significance of the co-evolution PCC. In brief, for the nonparametric test, first we permuted the protein sequences and the PWMs 1000 times to generate a background pool of the random sequences and the PWMs which have the same compositions and lengths as the original ones. Then, the real PCC was rank

tested against a null distribution of PCCs which were computed from the permuted proteins and PWMs. For the parametric test, we converted the real PCC to a test statistics following a t-distribution, followed by a two-tailed test against the null hypothesis that PCC=0.

2.3 Control for the effects of speciation

It is worth to note that each of the RRM-FL, RRM-RBR, KH-FL, and KH-RBR set contains proteins from multiple species. The observed correlation could be simply due to speciation. If it is true, we should see similar correlations between the KH's PWMs and RRM's RBPs or RRM's PWMs and KH's RBPs from the same distribution of species. Also, as we observe from Table 3 in the main paper, KH-RBR and RRM-RBR did not show statistically significant PCCs in the parametric test. Are these correlations really strong or not? For these purposes, we calculated the PCCs between the randomly paired RRM's PWMs and KH's protein sequences, and vice versa, keeping species the same, as suggested in [2]. The PCCs from both shuffled sets were much smaller. And the PCC values in Table 3 always ranked the highest in a 1000 times random shuffling pool (Supplementary Figure S1). The tests suggested that the correlations in Table 3 were not due to speciation, and they are strong.

3 The importance of sequence or structure preference alone

To assess the importance of the sequence information alone, RCK provided the functionality of sequence mode (run the program with -q option) which ignored all input structural probabilities by setting them to uniform distribution instead. When applying this functionality to KNN-RCK, this was equivalent to taking our PWM computed sequence scores as features and fitting a nonlinear model to training data. Moreover, similar to RCK's native sequence mode, KNN-RCK had the functionality of structure mode which ignored all sequence information by simply setting the input PWM to uniform distribution at each position (i.e. 0.25 for all entries in the matrix). These modes provided the flexibility for us to separately examine the importance of the sequence or structure information alone in RBP binding events. We demonstrate this in Supplement Figure S2, using two datasets: a subset of the InVitro dataset and the InVivoRay dataset. The subset of the InVitro dataset contained all the overlapped entries between the full InVitro dataset and the InVivoRay dataset.

As shown in Supplement Figure S2A, when evaluated on the InVitro dataset, the performance of structure alone (average PCC=0.409) was almost the same as the full model (0.417) with no statistically significant difference. While the performance of sequence mode (0.368) was significantly worse than the full model (p-value=0.022), and also worse than structure mode (p-value=0.050). However, when evaluated on the *in vivo* InVivoRay dataset (Supplement Figure S2B), the performance of sequence mode (average AUC=0.755) was better than structure mode (0.652), and was even better than the full model (0.667). These results were indeed consistent with our observations in Table 4 and Figure 2 that models using structure features did not perform as well as sequence models on the InVivoRay dataset, because the training data were short RNA probes which had weak structures and the testing data were long RNA segments which formed much more structures (i.e. the training and testing data were not quite identically distributed). Nevertheless, if in the future we have more structured RNA data so that we can train on them, then we may see better performance when incorporating structure information to the model.

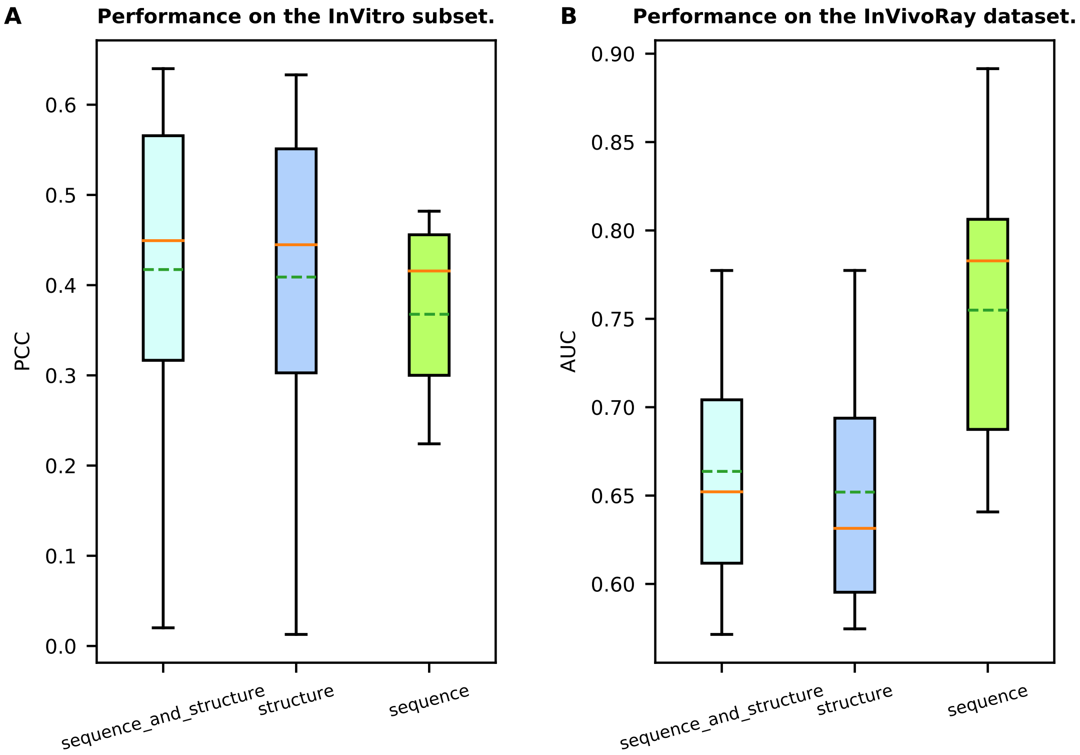

Figure S2: **Performance comparison with the full sequence-and-structure, structure alone, and sequence alone models.** The figure shows the comparison of using the full sequence-and-structure, structure alone, and sequence alone models in KNN-RCK, regarding performance in predicting *in vitro* binding on the InVitro subset and *in vivo* binding on the InVivoRay dataset. (A) Boxplot of *in vitro* performances in terms of PCCs. The dashed green lines in each box denote the mean values, and the brown lines denote the medians. Each model was trained on setA and tested on setB on the InVitro RNAcompete probes. (B) Boxplot of *in vivo* performances in terms of AUCs. Each model was trained on the entire InVitro RNAcompete dataset and tested on the InVivoRay CLIP/RIP dataset.

References

[1] Ray, D., Kazan, H., Cook, K.B., Weirauch, M.T., Najafabadi, H.S., Li, X., Gueroussov, S., Albu, M., Zheng, H., Yang, A., Na, H., Irimia, M., Matzat, L.H., Dale, R.K., Smith, S.A., Yarosh, C.A., Kelly, S.M., Nabet, B., Mecenas, D., Li, W., Laishram, R.S., Qiao, M., Lipshitz, H.D., Piano, F., Corbett, A.H., Carstens, R.P., Frey, B.J., Anderson, R.A., Lynch, K.W., Penalva, L.O.F., Lei, E.P., Fraser, A.G., Blencowe, B.J., Morris, Q.D., Hughes, T.R.: A compendium of rna-binding motifs for decoding gene regulation. Nature **499**(7457), 172–177 (2013). doi:10.1038/nature12311

[2] Yang, S., Yalamanchili, H.K., Li, X., Yao, K.-M., Sham, P.C., Zhang, M.Q., Wang, J.: Correlated evolution of transcription factors and their binding sites. Bioinformatics **27**(21), 2972–2978 (2011). doi:10.1093/bioinformatics/btr503

[3] Mahony, S., Auron, P.E., Benos, P.V.: Dna familial binding profiles made easy: Comparison of various motif alignment and clustering strategies. PLoS Computational Biology **3**(3), 61 (2007). doi:10.1371/journal.pcbi.0030061

[4] Pazos, F., Valencia, A.: Similarity of phylogenetic trees as indicator of protein–protein interaction. Protein Engineering **14**(9), 609–614 (2001). doi:10.1093/protein/14.9.609

[5] Larkin, M.A., Blackshields, G., Brown, N.P., Chenna, R., McGettigan, P.A., McWilliam, H., Valentin, F., Wallace, I.M., Wilm, A., Lopez, R., Thompson, J.D., Gibson, T.J., Higgins, D.G.: Clustal w and clustal x version 2.0. Bioinformatics **23**(21), 2947–2948 (2007). doi:10.1093/bioinformatics/btm404

[6] Schones, D.E., Sumazin, P., Zhang, M.Q.: Similarity of position frequency matrices for transcription factor binding sites. Bioinformatics **21**(3), 307–313 (2005). doi:10.1093/bioinformatics/bth480

[7] Mahony, S., Auron, P.E., Benos, P.V.: Dna familial binding profiles made easy: Comparison of various motif alignment and clustering strategies. PLoS Computational Biology **3**(3), 61 (2007). doi:10.1371/journal.pcbi.0030061

[8] Pietrokovski, S.: Searching databases of conserved sequence regions by aligning protein multiple-alignments. Nucleic Acids Research **24**(19), 3836–3845 (1996). doi:10.1093/nar/24.19.3836

[9] Hughes, J.D., Estep, P.W., Tavazoie, S., Church, G.M.: Computational identification of cis-regulatory elements associated with groups of functionally related genes in saccharomyces cerevisiae1. Journal of Molecular Biology **296**(5), 1205–1214 (2000). doi:10.1006/jmbi.2000.3519
